# Electrocatalytic and structural properties and computational calculation of PAN-EC-PC-TPAI-I_2_ gel polymer electrolytes for dye sensitized solar cell application

**DOI:** 10.1039/d1ra01983j

**Published:** 2021-06-29

**Authors:** Faisal I. Chowdhury, Jahidul Islam, A. K. Arof, M. U. Khandaker, Hossain M. Zabed, Ibrahim Khalil, M. Rezaur Rahman, Shahidul M. Islam, M. Razaul Karim, Jamal Uddin

**Affiliations:** Nanotechnology and Renewable Energy Research Laboratory (NRERL), Department of Chemistry, University of Chittagong Chittagong-4331 Bangladesh faisal@cu.ac.bd faisal.cubd@yahoo.com; Center for Ionics University of Malaya, Department of Physics, University of Malaya 50603 Kuala Lumpur Malaysia akarof@um.edu.my; Center for Radiation Sciences, Institute for Healthcare Development, Sunway University 47500 Subang Jaya Malaysia; School of Food and Biological Engineering, Jiangsu University Zhenjiang 212013 Jiangsu China; Nanotechnology and Catalysis Research Centre, Institute for Advanced Studies, University of Malaya 50603 Kuala Lumpur Malaysia; Department of Chemical Engineering and Energy Sustainability, Faculty of Engineering, University Malaysia Sarawak Malaysia; Department of Chemistry, University of Illinois at Chicago Chicago USA; Faculty of Engineering, University of Malaya 50603 Kuala Lumpur Malaysia; Center for Nanotechnology, Department of Natural Sciences, Coppin State University Baltimore MD USA

## Abstract

In this study, gel polymer electrolytes (GPEs) were prepared using polyacrylonitrile (PAN) polymer, ethylene carbonate (EC), propylene carbonate (PC) plasticizers and different compositions of tetrapropylammonium iodide (TPAI) salt. Linear sweep voltammetry (LSV) and electrochemical impedance spectroscopy (EIS) measurements were done using non-blocking Pt-electrode symmetric cells. The limiting current (*J*_lim_), apparent diffusion coefficient of triiodide ions 
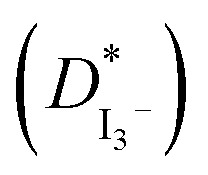
 and exchange current were found to be 12.76 mA cm^−2^, 23.41 × 10^−7^ cm^2^ s^−1^ and 11.22–14.24 mA cm^−2^, respectively, for the GPE containing 30% TPAI. These values are the highest among the GPEs with different TPAI contents. To determine the ionic conductivity, the EIS technique was employed with blocking electrodes. The GPE containing 30% TPAI exhibited the lowest bulk impedance, *R*_b_ (22 Ω), highest ionic conductivity (3.62 × 10^−3^ S cm^−1^) and lowest activation energy. Fourier transform infrared (FTIR) spectroscopy and X-ray diffraction (XRD) techniques were utilized for structural characterization. Functional group interactions among PAN, EC, PC and TPAI were studied in the FTIR spectra of the GPEs. An up-shift of the XRD peak indicates the polymer–salt interaction and possible complexation of the cation (TPA^+^ ion) with the lone pair of electrons containing site –C≡N at the N atom in the host polymer matrix. On the other hand, computational study shows that TPAI-PAN based GPE possesses the lowest frontier orbital bandgap, which coincided with the enhanced electrochemical and electrocatalytic performance of GPE. The dye-sensitized solar cell (DSSC) fabricated with these GPEs showed that the *J*_SC_ (19.75 mA cm^−2^) and *V*_OC_ (553.8 mV) were the highest among the GPEs and hence the highest efficiency, *η* (4.76%), was obtained for the same electrolytes.

## Abstract 2

In this study, gel polymer electrolytes (GPEs) were prepared using polyacrylonitrile (PAN) polymer, ethylene carbonate (EC), propylene carbonate (PC) plasticizers and different compositions of tetrapropylammonium iodide (TPAI) salt.

## Graphical abstract

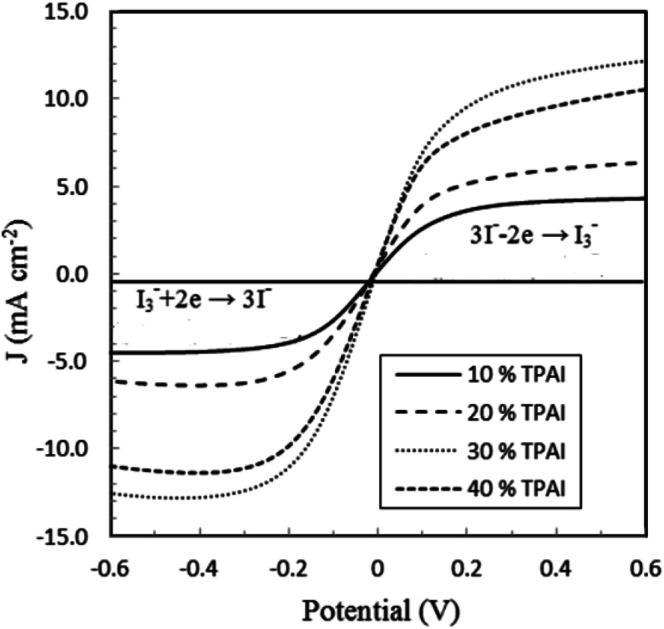

## 1. Introduction

One of the important components of dye-sensitized solar cells (DSSCs) is the electrolyte. The conducting polymers (CPs) have been regarded as alternative materials for DSSCs and other electronic devices because of their outstanding electrochemical properties, high electrical conductivity, high tensile strength, good stability and safety, ease of shaping, good processing ability, high flexibility, no spillage and low-costs.^1–7^ Due to the outstanding benefits of CPs, various types of polymer electrolytes (PEs) have been studied for many years. Nowadays, there are diverse families of conventional polymer electrolytes, such as gel polymer electrolytes, ionic rubber forms of polymer electrolytes and polyelectrolytes.^8^ There are a variety of traditional polymer based materials on or after synthetic polymers and their blends to biopolymer.^8^ Some of the well known polymers are polyacrylonitrile (PAN),^9–12^ polyethylene oxide (PEO),^13^ polyethylene glycol (PEG),^14,15^ poly(methyl methacrylate) (PMMA),^16,17^ poly(ethylene glycol) methyl ether methacrylate (PEGMA),^18^ poly(vinyl chloride) (PVC),^19^ poly(vinylidene fluoride) (PVdF)^20^ and poly(vinylidene fluoride-*co*-hexafluoropropylene) (PVdF-HFP).^21,22^ Until now, polysaccharides and modified polysaccharides based materials were in reality for their superior ionic conductivity at room temperature, which includes, for example, chitosan,^23,24^ cellulose^25^ and carrageenan.^26^ Recently, Di Noto *et al.* reported another two main kinds of hybrid inorganic–organic PEs that are mono-phase and multi-phase PEs.^27^ Also, various inorganic filler were also reported, such as titania (TiO_2_), zircronia (ZrO_2_), carbon-nanotubes (CNT), graphene (Gr) and silica (SiO_2_).^28–32^ In spite of these substantial amounts of research works PEs, their application in DSSCs is still limited.

The advantage of coordinating polymer and an appropriate salt in the solid-state have been used by researchers.^33^ Nevertheless, viscous electrolytes can limit ionic transport^34^ and penetration of the electrolyte into the mesoporous titania photoelectrode.^35^ The DSSCs fabricated with pure polymer electrolytes show lower values of short-circuit current, fill factor and efficiency when compared to DSSCs assembled with liquid electrolytes. To overcome these shortcomings of solid polymer electrolytes, researchers have considered liquid electrolytes, gel polymer electrolytes or quasi-solid electrolytes for DSSC application for their higher electrical conductivity and excellent stability.^36–38^ The conventional liquid electrolytes have serious defects, such as electrolyte evaporation, leakages, desorption, photo-degradation of the dye and corrosion of the platinum secondary electrode.^39–41^ Enormous efforts have been made to find alternatives for the liquid electrolytes, solid, quasi-solid/GPEs and ionic liquid blended electrolytes were studied to replace the liquid electrolytes.^42^ Also, for sustainable DSSC development, eco-friendly and cost-effective electrolyte and electrode materials are essential.^43^

Polymer electrolytes have demonstrated advantages over liquid electrolytes, including high tensile strength, better safety, ease of shape based fabrication, high processing ability, intact interfacing properties between electrodes, good flexibility, and no spillage.^8,44^ Very recent study has demonstrated that PAN, PEO, PVDF, PVC, polyurethane (PU) *etc.* based PEs have led to develop more efficient and stable DSSCs.^45,46^ Such a PEO based polymer material, *i.e.* PMMA, has been utilized to form GPE using different ratios of liquid electrolytes, which resulted improved efficiency (11.32%) in comparison with the available PEO based PEs.^47^ A PVA based GPE was applied in DSSC (natural dye-sensitized) that showed 2.62% efficiency.^48^ Buraidah and co-workers reported a high efficient (*η* = 9.61%) DSSC (N3 dye-sensitized) prepared with functionalized chitosan (phthaloylchitosan) based GPEs.^24^ Gohel and co-workers combined liquid electrolyte and gelator for enhancing cell performances, as it can increase ionic conductivity with the support of PEO-PMMA polymer hosted in EC/PC/THF plasticizer/solvent salt complexes containing I^−^/I_3_^−^ redox shuttle.^49^ Likewise, Nair *et al.*^50^ reported superior ionic conductivity in protic ionic liquids (PILs) doped in acidic medium (*i.e.* glacial acetic acid) with TBP in GPE. In the twenty-first century, researchers focused again on investigating aqueous systems in DSSCs by replacing organic solvents.^51–54^ The influential work was reported by O'Regan *et al.* in 2010 that might be unquestionably the original work for the scientific community, in which they used different ratios of methoxy propionitrile (MPN)-water electrolytes.^55,56^ In another work on water based DSSC showed enhanced photocurrent densities and photovoltages, which resulted higher efficient solar cells except observing lower fill factors.^57^

In GPEs, a high amount of organic solvent is trapped in the polymer matrix, resulting in the compensation of solvent leakage and volatilization problems. GPEs have good contact with electrodes,^58^ higher ionic conductivity than solid polymer electrolytes,^59^ rationally high photovoltaic performance and better thermal and mechanical stability over liquid electrolytes.^60^ Classical GPE contains small portions of the low molar mass polar polymer matrix in large amount of organic plasticizer (ethylene carbonate, EC and/or propylene carbonate, PC) with polar aprotic organic solvents (acetonitrile, AN and tetrahydrofuran, THF). The plasticizer lowers the glass transition temperature of the polymer by introducing disorders in the crystalline phase, increasing its segmental mobility and free volume of the system. Even though GPEs have many advantages, their electrical and photovoltaic performance is still far away for considering them in the photovoltaic application commercially due to some limitations. According to scientific reports,^61–63^ the transportation of charge carriers is hindered by the gel polymer network inside the polymer matrixes and gelators may interact or even react with chemical compounds of the electrolytes.

PAN based electrolytes have also been extensively studied because of their good ionic conductivity, excellent chemical and flame resistance, electrochemical stability.^64–67^ PAN is one of the most valuable fiber-forming polymers and is extensively used due to its high abrasion resistance, strength and good insect resistance.^68^ It is used to produce a large variety of products, including ultrafiltration membranes, hollow fibers for reverse osmosis, fibers for textiles, oxidized flame retardant fibers and carbon fiber. However, the conductivity of pristine PAN is low (<10^−14^ S cm^−1^) that restricts further applications. Triiodide/iodide redox couple in GPEs is important for the DSSC operation, which is formed by the iodide salt and iodine. The concentration and size of the salt have significant roles in the photovoltaic DSSC performance. It has been observed that the photocurrent drops and the photovoltage rises with increasing radius of the cations.^69^ This is because the conduction band energy of TiO_2_ and the associated influence on the electron injection efficiency vary with the cation nature.^70^ According to reports,^71,72^ smaller cation (Li^+^, Na^+^, Mg^2+^) speeds up the dye regeneration. Furthermore, researchers have revealed that the cation size of the doping salt plays an important role in the improvement of iodide conductivity. Several researchers have argued that larger cations enhance the iodide ion mobility, resulting in better DSSC performance,^73,74^ and DSSCs based on GPEs with salts containing larger cations like tetrapropyl ammonium iodide (Pr_4_NI) and tetrahexyl ammonium iodide (Hex_4_NI) have been reported.^75–77^

The dynamics of electro-catalysis of iodide/triiodide redox mediator on cathode or counter electrode is one of the most critical phenomena in DSSC operational mechanism. The counter electrode reduces triiodide (I_3_^−^) into iodide (I^−^) to regenerate the light-absorbing sensitizer after electron injection.^77^ Optimization of I_2_ concentration is also important because if the iodine concentration is too high, polyiodide species like I_5_^−^, I_7_^−^ and I_9_^−^ may also be formed, but in particular, only triiodide seems to be of importance in DSSC electrolytes.^78^ The limiting current, exchange current and charge transfer resistance are also vital parameters for the DSSCs optimization.^79^

According to the very recent review article published in 2020 by Teo *et al.*^80^ on polyacrylonitrile polymer host based GPEs for DSSCs application, PAN-EC-PC-TPAI-I_2_ gel polymer electrolytes were reported to be promising for DSSC^74,81–88^ In this context, PAN-EC-PC-TPAI-I_2_ gel polymer electrolytes were investigated in the present work, which included various characterizations, such as electrocatalytic performance, detailed vibrational study and XRD analysis. Also, we are reporting the computational calculation (frontier orbitals, HOMO–LUMO energy states, *etc.*) of the PAN-EC-PC-TPAI-I_2_ based GPEs systems for the first time. Finally, the prepared GPE with maximum conductivity was applied in DSSC.

## 2. Experimental

### 2.1. Materials

Polyacrylonitrile (PAN), ethylene carbonate (EC), propylene carbonate (PC), quaternary ammonium iodide salt: tetra-propylammonium iodide (TPAI), and iodine (I_2_) were procured from Aldrich. The purity for all starting materials was greater than 98%. Table 1 shows the chemical structures of PAN, EC, PC and TPAI. Prior to use PAN and TPAI were vacuum dried for 24 h at 50 °C in a vacuum oven. Other materials were used as received. Conducting glass substrates (fluorine doped tin oxide, FTO) with sheet resistance of 10 Ω cm^−2^, sensitizing N_3_ dye [*cis*-bis(isothiocyanato)bis(2,2′-bipyridyl-4,4′-dicarboxylato ruthenium(ii)] and platinum catalyst solution (plastisol) were purchased from Solaronix SA, Switzerland. TiO_2_, P90 (14 nm) and P25 (21 nm) were purchased from AEROXIDE.

**Table 1:** Chemical structures of PAN, EC, PC and TPAI

Chemicals	Chemical formula	Chemical structures	Company
Polyacrylonitrile (PAN)	[–CH_2_–CH(CN)–]_*n*_	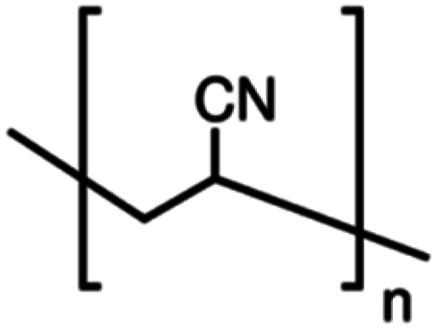	Sigma-Aldrich
Ethylene carbonate (EC)	(CH_2_O)_2_CO	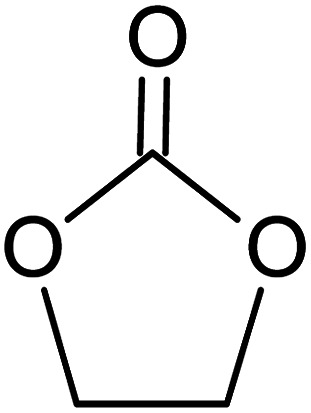	Sigma-Aldrich
Propylene carbonate (PC)	CH_3_C_2_H_3_O_2_CO	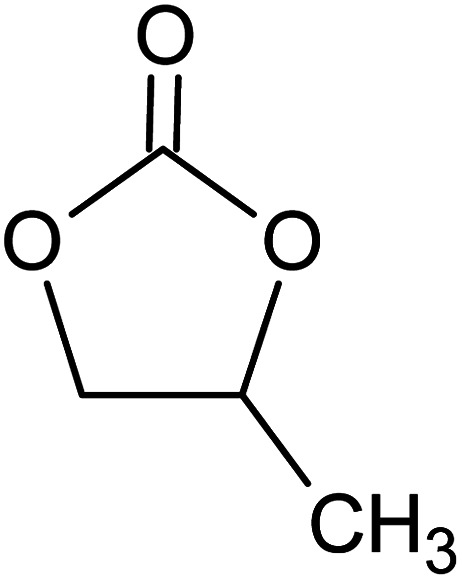	Sigma-Aldrich
Tetrapropylammonium iodide (TPAI)	(CH_3_CH_2_CH_2_)_4_^+^I^−^	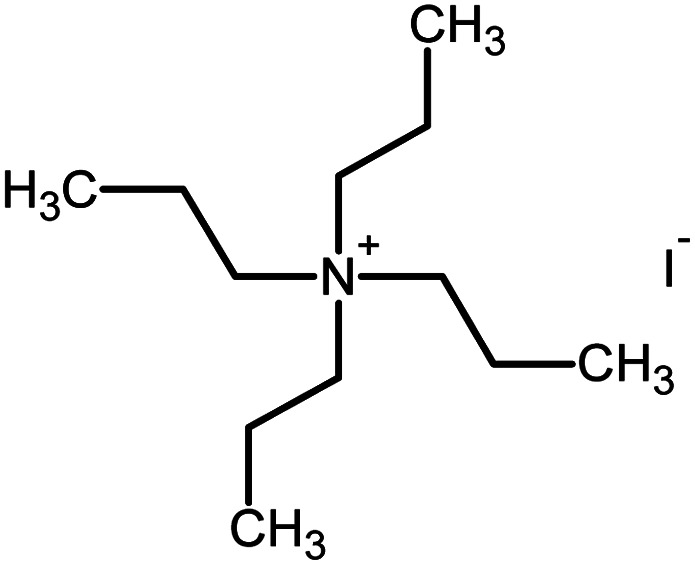	Sigma-Aldrich

### 2.2. Gel polymer electrolyte (GPE) preparation

For the preparation of gel polymer electrolytes, PAN, EC and PC were used as host polymer and plasticizers, whereas I_2_ was used to form the redox mediator. The GPEs were prepared following the composition PAN : EC : PC : *x*TPAI : *y*I_2_, where *x* is 10, 20, 30 and 40 wt% with respect to the PAN/EC/PC mass and *y* is 10 mol% of TPAI. Table 2 shows the compositions of the GPEs. The masses of PAN, EC and PC were kept at 0.4, 1.5 and 1.5 g, respectively. EC and PC were mixed and stirred in a glass bottle and heated at about 110–120 °C. PAN polymer was then added with continuous stirring and heating. After a homogenous solution was obtained, TPAI salt was added to the solution and stirred. The I_2_ was added to the mixture to produce I^−^/I_3_^−^ redox mediator. The stirring was continued to get a homogenous and gelatinized mixture. The final GPEs were used for characterization and application in DSSCs.

**Table 2:** Compositions of PAN based GPEs

% TPAI	PAN (g)	EC (g)	PC (g)	TPAI (g)	I_2_ (g)
10	0.4	1.5	1.5	0.50	0.034
20	0.4	1.5	1.5	1.00	0.069
30	0.4	1.5	1.5	1.50	0.103
40	0.4	1.5	1.5	2.00	0.137

### 2.3. Characterization

#### 2.3.1 Linear sweep voltammetry: diffusion coefficient of I_3_^−^

The linear sweep voltammetry (LSV) technique was applied to measure the apparent diffusion coefficient of triiodide (I_3_^−^) ion, 
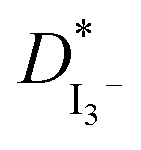
. The symmetrical thin-layer dummy cell with 53 μm thickness was used for the measurement of limiting current (steady-state current) densities; the cell was constituted of two platinized counter electrodes separated by the Scotch tape with size of 53 μm.^86,87^ The applied voltage was swept from −0.6 V to 0.6 V with the slow rate of 10 mV s^−1^ and 
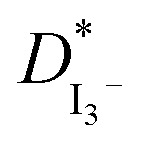
 was determined by measuring the diffusion-limited current, *J*_lim_. The experiment was done in triplicate. The electrochemical reaction at the Pt/electrolyte was on interface due to the application of potential followed by the eqn (1),

1 I_3_^−^ + 2e^−^ ⇌ 3I^−^

#### 2.3.2 Electrochemical impedance spectroscopy (EIS)

Impedance measurements for PAN-EC-PC-*x*TPAI-*y*I_2_ GPEs were performed using the HIOKI 3532-50 LCR Hi-Tester in the frequency range from 50 Hz to 1 MHz from 25 °C to 100 °C, where *x* = 0%, 10%, 20%, 30% and 40% and *y* is the required amount of I_2_. To measure the current, a small sinusoidal potential was applied through the samples. The applied voltage was 10 mV. The GPE of 2 cm diameter was sandwiched between two stainless-steel electrodes. The Nyquist plots were drawn as negative imaginary impedance *versus* real impedance. The bulk resistance, *R*_b_, was acquired from the intercept of the Nyquist plot to the real impedance axis. The following equation was used to calculate the electrical conductivity, *σ*, of the samples:^88^2
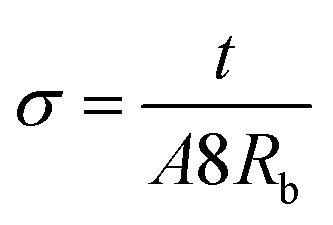
where *t* is the sample thickness and *A* is the electrode–electrolyte contact area. This test was done according to ASTM G106-89.^89^ Triplicate measurement was performed for all the experiments.

#### 2.3.3 Fourier transform infrared (FTIR) spectroscopy

IR spectra for the GPEs of various amounts of TPAI were obtained using a Thermo Scientific model Nicolet iS10 FTIR spectrometer. The spectra were recorded in the transmittance mode and then converted to absorbance mode between 650 and 4000 cm^−1^ at 4 cm^−1^ resolutions at ambient temperature. Background spectrum was recorded prior to the capture of the IR spectrum for every sample run. The test was run according to ASTM E168-16 (ref. 90) and ASTM E1252-98.^91^

#### 2.3.4 X-ray diffraction (XRD)

XRD diffractograms were collected for each sample for the structural characterization. Measurement of each sample was performed in the 2*θ* angle between 5° and 45° using an Olympus BTX Benchtop diffractometer and 250 scans were recorded for each sample. ASTM D5380-93 (ref. 92) was referred and used as a guideline for the XRD experiment.

## 3. Results and discussion

### 3.1. Linear sweep voltammetry at symmetrical cell: diffusion coefficient of I_3_^−^

Linear sweep voltammetry, as well as cyclic voltammetry, is a potential technique to characterize the electrocatalytic activity of electrocatalysts.^93^Fig. 3 shows the characteristic linear sweep voltammetry (LSV) curves for the electrolyte systems containing different compositions of TPAI. The current densities attain saturations for both polarities at above 0.3 V. The anodic and cathodic limiting current plateaus were relatively similar, which indicates the steady-state equilibrium conditions. It was noted that the limiting current for triiodide ions acts as iodide concentration, which showed greater concentration compare with I_2_.^94^ Hence, limiting current densities (*J*_lim_) can only be used to determine the apparent diffusion coefficient of triiodide, 
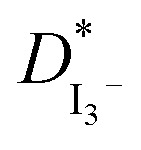
, according to the following relation:3
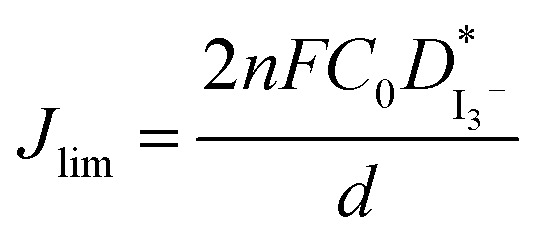
where *n* = 2 is the electron number required for the reduction of triiodide to iodide, *C*_0_ is the initial concentration of the triiodide ions, *d* the thickness of the cell and *F* the Faraday constant.

The *J*_lim_ and 
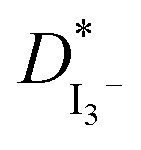
 values for TPAI containing GPE systems are tabulated in Table 4. The value of 
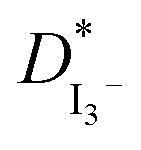
 increased with the increased I_2_ content and it was highest at 5.5 × 10^−7^ cm^2^ s^−1^ for 0.051 (g) I_2_ containing electrolyte with TPAI = 30 wt%. The values of 
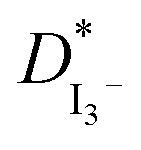
 decreased if more I_2_ was added. This is because excessive ions can hinder ion diffusion. Similar behaviour was also observed for the conductivity of these electrolytes. However, more I_2_ can produce more I_3_^−^ ions, which may cause ion aggregation and/or micellization and results in a lower diffusion rate of I_3_^−^ ions. In addition, more salt provides more ions in the electrolyte, which may reduce the volume of free space, causing lower diffusion.

### 3.2. Exchange current density, *J*_0_

For EIS experiments, the electrolyte composition used corresponds to the electrolyte composition for the DSSC. The EIS measurements have been performed using symmetric cells fabricated with two identical Pt–electrodes^95^ under conditions that closely simulate the DSSC since adsorption of the electrolyte components on Pt could modify the kinetics of the I^−^/I_3_^−^ reaction. The *R*_ct_ associated with the equilibrium of eqn (1) is a measurement of the electro-catalytic activity for the tri-iodide (I_3_^−^)/iodide (I^−^) redox reaction.

For all the four investigated GPEs, the Nyquist plots showed two semicircles: the left one was for the higher frequency region and the right one was for the lower frequency region. The high frequency intercept along the real axis represents the ohmic series resistance (*R*_s_).^93^ The semicircle in the region of high frequency corresponds to the charge-transfer process (*R*_ct_) of electrolyte/electrode interface, whereas the semicircle represents the low frequency region. It was due to the Nernst diffusion process of triiodide ions.^96^ As shown in Fig. 3 and Table 3, it can be observed that *R*_s_ value was the smallest for 30% TPAI GPE because of its superior electrical conductivity, which revealed the improvement of DSSCs performance. Furthermore, the charge-transfer resistance *R*_ct_ values for the 10%, 20%, 30% and 40% TPAI containing GPEs were calculated to be 10.00, 9.20, 3.80 and 5.10 Ω, respectively (Table 4). The smallest *R*_ct_ (3.80 Ω) value indicates that the 30% TPAI GPE had a superior electrocatalytic activity compared to other GPEs.^97^

**Table 3:** Limiting current or steady-state current (*J*_lim_), diffusion coefficients of I_3_^−^ ion 
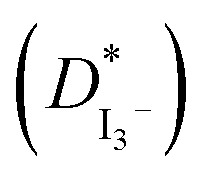
, charge-transfer resistance (*R*_ct_) and exchange current density (*J*_0_) of GPEs containing different composition of iodine. *J*_0,EIS_ and *J*_0,Tafel_ have been calculated from EIS and Tafel polarization curves, respectively

TPAI	10%	20%	30%	40%
I_2_ (g)	0.018	0.035	0.051	0.069
*J*_lim_ (mA cm^−2^)	4.46	6.32	12.76	11.29
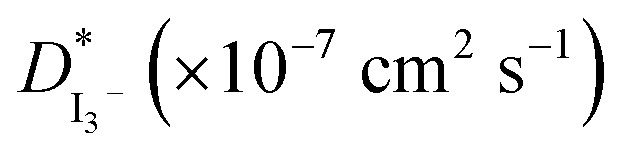	9.15	12.23	23.41	19.67
*R*_S_ (Ω)	22.60	21.50	20.40	20.60
*R*_ct_ (Ω)	10.00	9.20	3.80	5.10
*R*_diff._ (Ω)	20.20	20.00	14.60	19.00
*J*_0,EIS_ (mA cm^−2^)	5.41	5.88	14.24	10.61
*J*_0,Tafel_ (mA cm^−2^)	3.98	5.62	11.22	10.00

**Table 4:** Bulk impedance and conductivity for the GPEs with different TPAI content

TPAI	*R*_b_/ohm	Conductivity, *σ* (S cm^−1^)	Activation energy, *E*_a_ (eV)
0%	2900	2.74 × 10^−5^	19.94
10%	42.0	1.89 × 10^−3^	11.73
20%	36.0	2.21 × 10^−3^	11.11
30%	22.0	3.62 × 10^−3^	10.09
40%	30.0	3.26 × 10^−3^	11.12

The exchange current density, *J*_0_, *i.e.*, the equal cathodic and anodic currents normalized to the projected electrode area at equilibrium was calculated from *R*_ct_ by the following equation:4
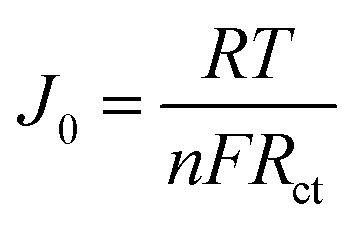
where *R* is the molar gas constant, *T* is the room temperature, *n* is the number of electrons involved in the redox reaction, *F* is the Faraday constant and *R*_ct_ is the kinetic component of the resistance determined by EIS multiplied by the projected area (*r* = 0.275 cm) of the electrode.

**Fig. 1:**
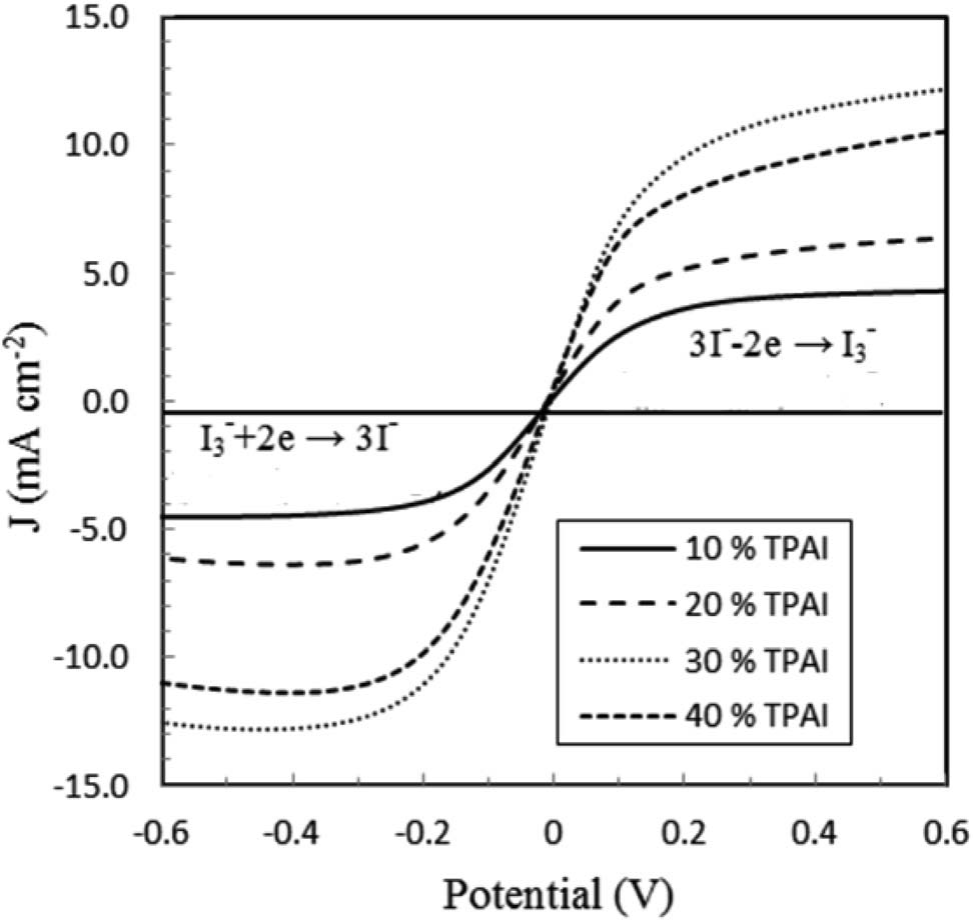
Linear sweep voltammograms (LSV) of GPEs at varying concentration of TBAI with Pt ultramicroelectrode. Scan rate: 10 mV s^−1^.

**Fig. 2:**
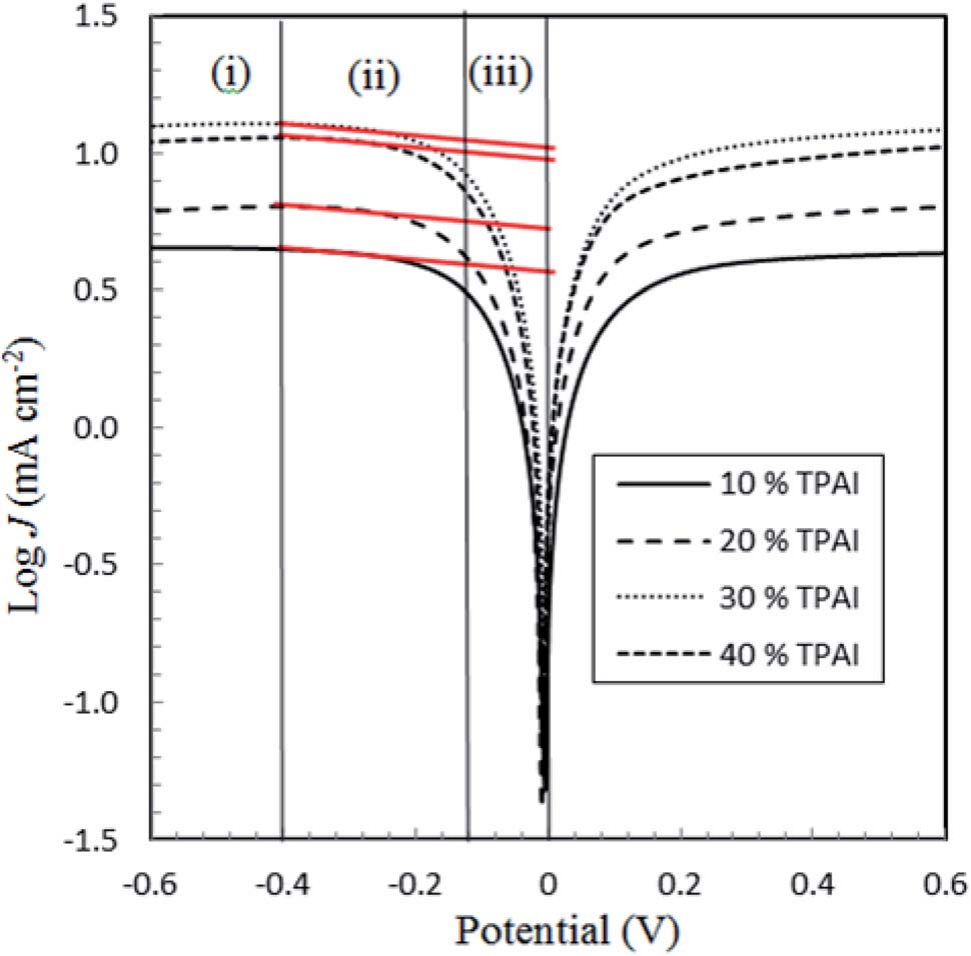
Tafel polarization curves for the electrolytes with different TPAI containing GPEs.

From the LSV measurements, the exchange current density, *J*_0_, has also been estimated using the Tafel polarization technique. The linear sweep voltammetry (LSV) curves obtained from symmetrical cells were converted to logarithmic current–voltage (log *J*–*V*) Tafel polarization curves (Fig. 1).^98^ Tafel curves had three zones: (1) polarization region (V < 120 mV), (2) Tafel zone (120 mV < 400 mV) and (3) diffusion zone (V > 400 mV),^98^ which shown in Fig. 4. *J*_0_ was obtained by extrapolating the anodic or cathodic curves in its Tafel zone and the cross point at 0 V, which is displayed in Table 3. The current exchange densities estimated from LSV were closer to those obtained from EIS measurement and show a similar variational trend. The values showed an increase with an increase in TPAI concentration. At 30% TPAI containing GPEs, the *J*_0_ value was the highest, indicating the best current/charge transferring ability, as well as the minimum over potential among the GPEs. The fast consumption of I_3_^−^*i.e.* high exchange current being the source of less energy loss resulting in good electrode–electrolyte catalytic activity and better cell performance because the electro-catalytic reduction of triiodide ions (I_3_^−^) on the surface of a CE is a rate-determining step in a DSSC.^99–101^ The GPE with 30% TPAI had the optimum I_2_ composition, confirming the best I^−^/I_3_^−^ electro-catalytic performance on Pt CE, which was dramatically reduced if more iodine was added in to the system. It was due to the formation of poly-iodides and ion aggregation (Fig. 2).^102^

**Fig. 3:**
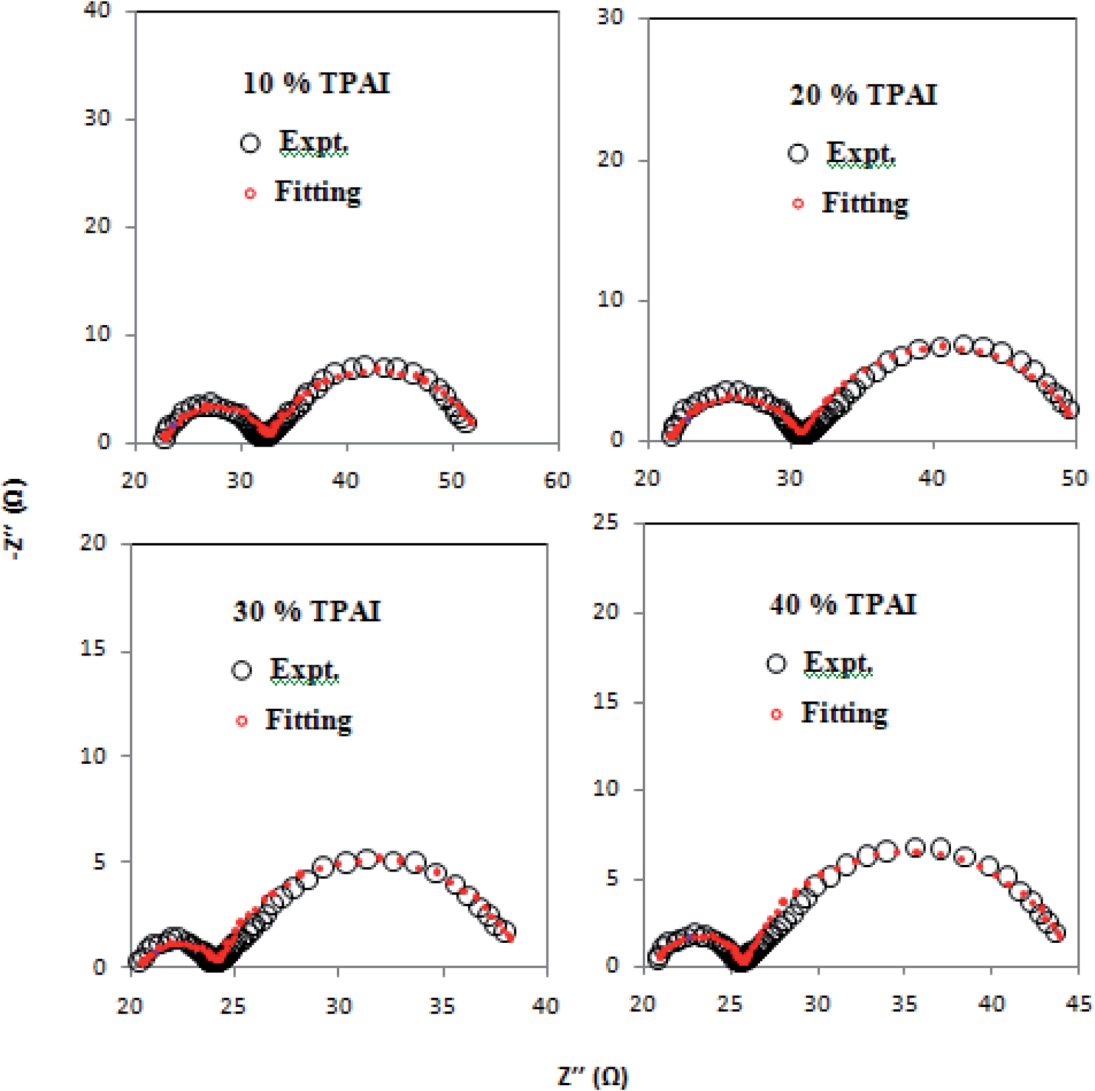
Nyquist plots of the dummy cells were fabricated with two identical Pt-ultramicroelectrodes with different percentages of TPAI salt containing GPEs.

**Fig. 4:**
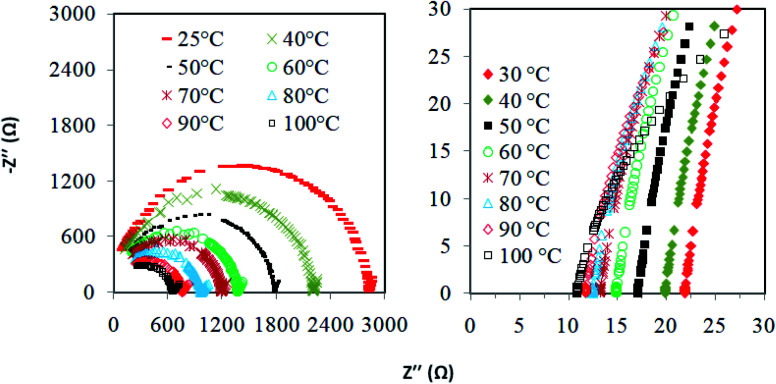
Nyquist plot for the PAN-EC-PC-TPAI-I_2_ GPE with (i) 0% and (ii) 30% TPAI.

### 3.3. Ionic conductivity measurements

Fig. 4 presents the Nyquist plots of imaginary impedance *versus* real impedance for PAN-EC-PC-TPAI-I_2_ GPEs with a varying weight percentage of TPAI (0% and 30%) at different temperatures. For 0 wt% TPAI, the Nyquist plots take the form of a semicircle and GPE with TPAI salt showed only a spike in their Nyquist plots. The occurrence of spike in the complex impedance plots may be ascribed to the accumulation of charges at the electrolyte–electrode (blocking electrode) interface, which is commonly described as the double layer capacitive effect (*C*_dl_).^102^ From the Nyquist plots, the bulk resistance, *R*_b_, was estimated and used to calculate ionic conductivity (*σ*) of the GPEs. Table 4 exhibits the thickness, *t*, *R*_b_ and *σ* for the GPEs. It was evident that the bulk impedance decreased with the increased percentage of TPAI salt, showing the lowest value of 22 Ω at 30% TPAI containing GPE. Consequently, *σ* increased with the increase in TPAI concentration and reached the highest value of 3.62 × 10^−3^ S cm^−1^ at 30% TPAI and then decreased with further addition of salt. It can be interpreted considering that in the initial stage the conductivity increases due to the addition of more ions in the polymer matrix until it reaches a maximum and after that ion recombination dominates all other processes favorable for conductivity.^103^

**Fig. 5:**
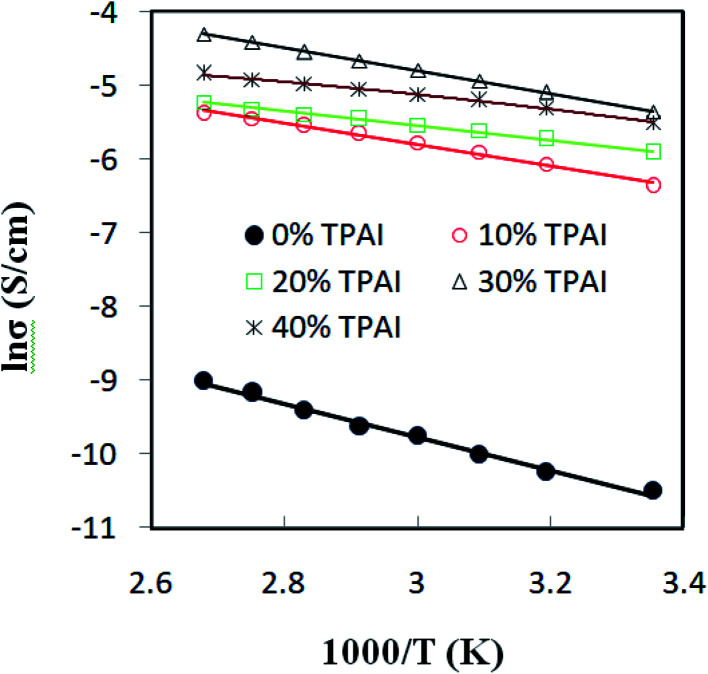
Conductivity (*σ* × 10^4^ (S cm^−1^)) *versus* temperature (*T*/K).

Fig. 5 shows ln *σ versus* 1000/*T* for the GPEs containing different percentages of TPAI. The later relation follows the Arrhenius equation of the following form:5
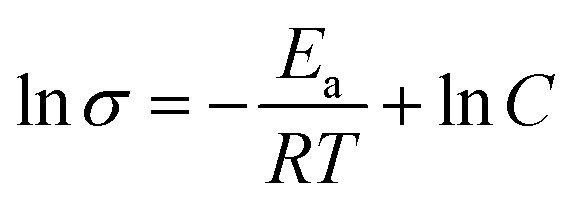
where *σ* represents ionic conductivity, *E*_a_ activation energy, *R* molar gas constant, *T* absolute temperature and *C* pre-exponential factor. The activation energy for transportation of ions decreased with TPAI percentage and it was the lowest for 30% TPAI containing GPE, which is conceivable with the conductivity behavior.

### 3.4. FTIR spectrophotometric analysis

**Fig. 6:**
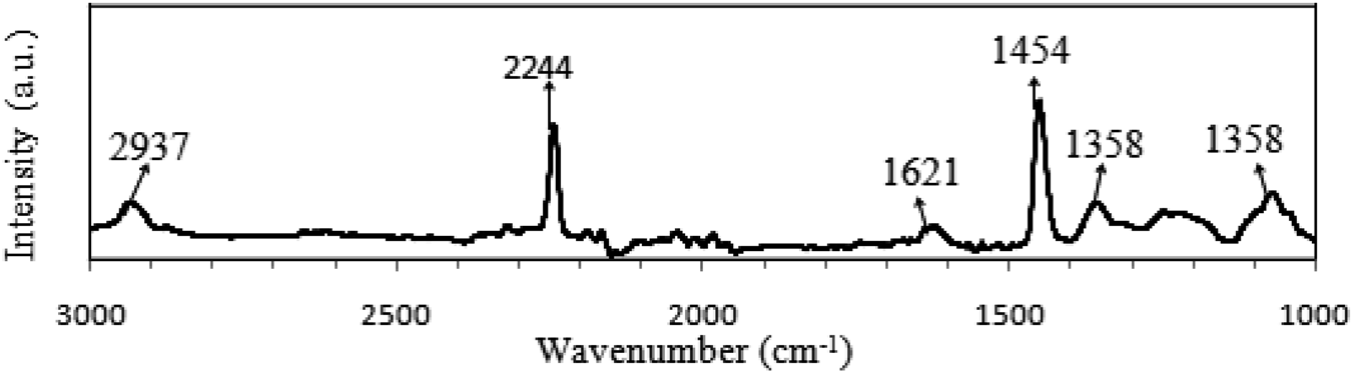
FTIR spectra of pure PAN powder.

FTIR spectrum of pure PAN is presented in Fig. 6 and the peaks assignments are shown in Table 5. For the pure PAN, the distinguishably sharp peak at 2244 cm^−1^ corresponds to –C≡N functional group stretching vibration.^104–106^ The C–H asymmetrical stretching vibration mode of –CH_2_– groups in PAN was observed at 2937 cm^−1^ as a broad peak in the spectrum.^107–110^ Another sharp peak at 1454 cm^−1^ represented the C–H bending of –CH_2_– groups in PAN.^105,107,108^ The combined vibration of C–H bending and wagging in CH and –CH_2_– groups was assigned at 1358 cm^−1^.^111,112^ A broad peak at 1073 cm^−1^ was assigned for the skeletal vibration, C–C symmetrical stretching of C–C≡N in PAN polymer.^111,113^ A peak at 1621 cm^−1^ was allocated for O–H bending of absorbed water.^114^

**Table 5:** FTIR peak assignment for PAN, EC, PC, TPAI and GPE

Component	Wavenumber (cm^−1^)	Assignments	Literature
PAN	2937	C–H asymmetrical stretching of –CH_2_– groups	107–110
	2244	C≡N stretching of –C≡N groups	104–106
	1621	O–H bending of the absorbed water	114
	1454	C–H bending of –CH_2_– groups	105, 107 and 108
	1358	C–H bending of CH groups + C–H wagging of –CH_2_–groups	111 and 112
	1073	C–C symmetrical stretching of C–CN	111 and 113
EC	2931	CH_2_ stretching	115
	1866	C=O stretching	64 and 115–117
	1484	CH_2_ scissoring/CH_2_ bending	115 and 116
	1420	CH_2_ wagging	64 and 115
	1392	CH_2_ wagging	64 and 115
	1218	CH_2_ twisting	115
	1218	CH_2_ twisting	115
	1158	CH_2_ twisting/skeletal stretching	115 and 116
	1071	Ring stretching/ring breathing	64 and 115
	970	Ring stretching/skeletal stretching	64 and 115
	891	Ring breathing	64 and 115
	770	CH_2_ rocking	115
	714	C=O bending/ring bending	115 and 118
PC	2931	CH_2_ stretching	115
	1781	C=O stretching	64, 116, 117, 119 and 120
	1485	CH_2_ scissoring/CH_2_ bending	116
	1388	CH_2_ wagging	64
	1175	CH_2_ twisting/skeletal stretching	116
	1117	COC asymmetrical vibration	121
	1071	Ring stretching/ring breathing	64
	1045	(CO_3_)^2−^ symmetric stretching vibration	122
	970	Ring stretching/skeletal stretching	116
	891	Ring breathing	64
	775	CH_2_ rocking	115
	710	C=O bending/ring bending	115 and 118
GPEs	2964	CH_2_ asymmetrical stretching vibrations (up-shifting from 2937 cm^−1^)	
	1789	C=O stretching (up-shifted from 1866 cm^−1^)	
	1772	C=O stretching (down-shifted from 1781 cm^−1^)	
	1480	CH_2_ scissoring/CH_2_ bending (down-shifted from 1485 cm^−1^)	
	1389	CH_2_ wagging (down-shifted from 1392 cm^−1^)	
	1354	C–H bending of CH groups + C–H wagging of –CH_2_– groups (down-shifted from 1358 cm^−1^)	
	1159	CH_2_ twisting/skeletal stretching (down-shifted from 1178 cm^−1^)	
	1118	C–C–C bending (up-shifted from 1109 cm^−1^)	
	1051	C–C symmetrical stretching of C–CN (down-shifted from 1073 cm^−1^)	

**Fig. 7:**
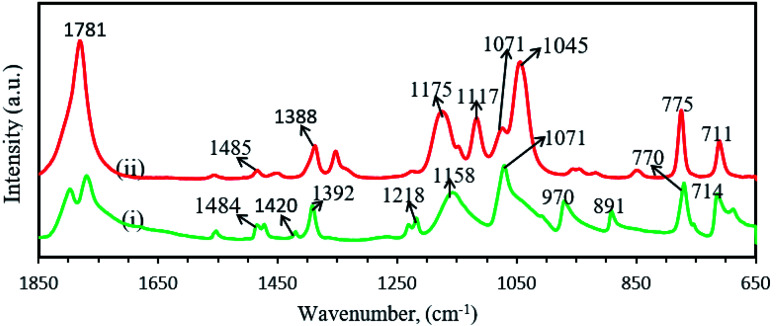
FTIR spectra of (i) EC and (ii) PC.

Fig. 7 shows the FTIR spectra of ethylene carbonate (EC) and propylene carbonate (PC) and corresponding peak vibrations are depicted in Table 5. The IR spectrum of EC contains a number of different modes of CH_2_ vibrations at different wave-numbers, such as stretching at 2931 cm^−1^,^115^ scissoring/bending at 1484 cm^−1^,^115,116^ wagging 1420 and 1392 cm^−1^,^64,115^ twisting at 1218 cm^−1^,^115^ twisting/skeletal stretching at 1158 cm^−1^.^115,116^ The small peak at 1866 cm^−1^ was assigned for C=O stretching vibration.^64,115–118^ The peaks at 1071, 970 and 891 cm^−1^ were designated for ring stretching/ring breathing, ring stretching/skeletal stretching and ring breathing, respectively.^64,115^ Rocking of CH_2_ and bending/ring bending of C=O were observed at 770 and 714 cm^−1^, respectively.^115,118^ The FTIR peaks of PC (Fig. 5) were nearly same as EC, except C=O stretching vibration at 1781 cm^−1^ (ref. 64, 116, 117, 119 and 120) and COC asymmetrical vibration at 1117 cm^−1^.^121^ The sharp peak at 1045 cm^−1^ was identified as (CO_3_)^2−^ symmetric stretching vibration.^122^

**Fig. 8:**
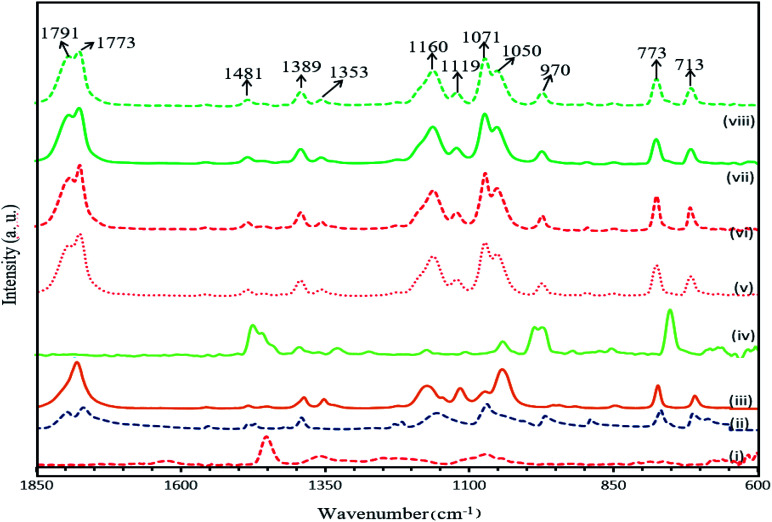
FTIR spectra of (i) PAN, (ii) EC, (iii) PC, (iv) TPAI, (v) 10% TPAI, (vi) 20% TPAI, (vii) 30% TPAI, (viii) 40% TPAI.

Fig. 8 shows the FTIR spectra for PAN, EC, PC, TPAI and 10%, 20%, 30% and 40% TPAI containing GPEs. In GPEs, the original peak 2937 cm^−1^ for CH_2_ asymmetrical stretching vibrations downshifts from 2964 cm^−1^, 1485 for CH_2_ scissoring/CH_2_ bending downshifts to 1480 cm^−1^, 1392 for CH_2_ wagging up-shifts to 1389 cm^−1^ and CH_2_ twisting/skeletal stretching downshifts from 1178 to 1159 cm^−1^. The C=O stretching mode of vibration at 1866 and 1781 cm^−1^ shifts to 1789 and 1772 cm^−1^, respectively. Furthermore, C–H bending (CH groups) and wagging (–CH_2_–) mode of vibration downshift from 1358 to 1354 cm^−1^. Similarly, C–C–C bending up-shift from 1109 to 1118 cm^−1^ and C–C symmetrical stretching of C–CN downshifts from 1073 to 1051 cm^−1^.

### 3.5. XRD analysis

**Fig. 9:**
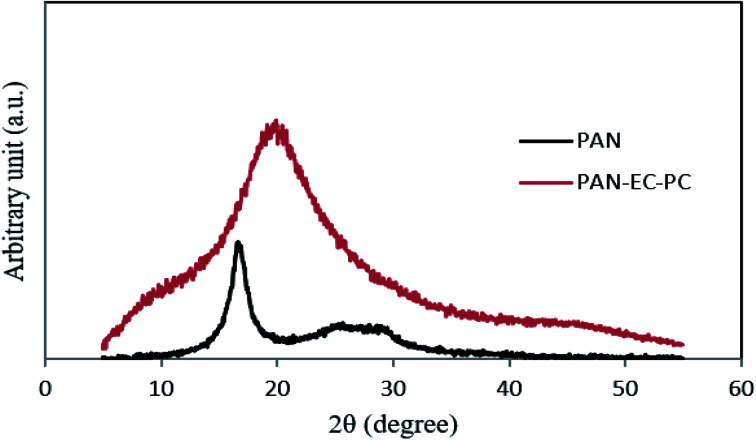
XRD pattern of PAN and PAN-EC-PC-0% TPAIGPE.

**Fig. 10:**
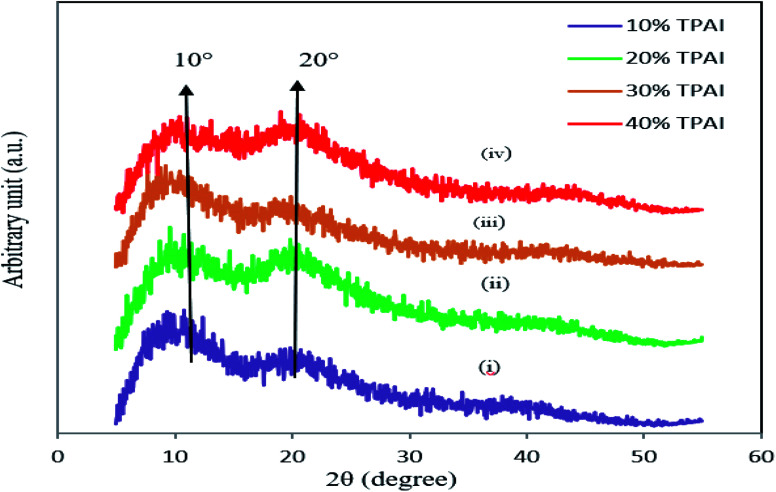
XRD pattern of (i) PAN-EC-PC-10% TPAI-I_2_, (ii) PAN-EC-PC-20% TPAI-I_2_, (iii) PAN-EC-PC-30% TPAI-I_2_ and (iv) PAN-EC-PC-40% TPAI-I_2_ GPEs.

To perform the structural characteristics of GPEs with different percentages of TPAI, X-ray diffraction studies were carried out. Fig. 9 exhibits the X-ray diffraction patterns of (i) PAN and (ii) PAN-EC-PC-0% TPAI GPE, respectively. Fig. 10 shows XRD pattern of PAN-EC-PC-*x*TPAI-I_2_ GPEs where *x* stands for 10%, 20%, 30% and 40%. Based on the equatorial reflections in diffraction patterns of PAN,^123,124^ it can be concluded that PAN had only two-dimensional order without periodicity along the chain axis. Therefore, PAN is a paracrystalline or laterally ordered polymer. PAN crystals usually show two diffraction peaks at 2*θ* ≈ 17 and 29°.^125^ According to the literature, orthorhombic lattice describes the crystal structure of PAN whereas dry PAN has hexagonal lattice.^126–128^ The diffraction patterns were also indexed as (010) and (300) at 2*θ* ≈ 17 and 29°, respectively, on the basis of hexagonal packing of PAN molecules.^129,130^ However, the XRD pattern of the pure PAN has semi-crystalline structure and the crystalline peak at 2*θ* ≈ 17° corresponds to orthorhombic (110) reflection.^131–133^ The addition of salt (TPAI) into PAN matrix results in a significant reform of XRD pattern observed in terms of (1) a systematic shifting and enlargement of the main peak (2*θ* ≈ 17°) of pure PAN toward a higher angle (2*θ* ≈ 20°) in PAN-EC-PC GPE and (2) generation of new peaks at 2*θ* ≈ 10° and 20° for 10%, 20%, 30% and 40% TPAI containing GPEs, which shown in Fig. 10. There was an up-shifting of XRD peak due to the increase in *d*-spacing of the polymer matrix, which is the evidence for polymer–salt interaction and complexation of cation (TPA^+^ ion) with lone pair electron containing site (–CN) in the host polymer matrix. Furthermore, the addition of TPAI containing long propyl chain (CH_3_–CH_2_–CH_2_–) prevents polymer chain reorganization causing significant disorder in the polymer chains that promotes the interaction between them. TPA^+^ ions may break the regular arrangement of PAN polymer backbone and aggregate through non-polar hydrophobic chain initiated micellization, which severely disturbs the order of crystalline phase of polymer causing development of amorphousness in the GPEs. Furthermore, microcrystalline arrangements create body centered cubic (BCC), *Im*3*m* structure in GPE on dye-TiO_2_ surface that may contain nanochannels promoting migration/conduction of ion results enhanced ionic conductivity.^134,135^

### 3.6. Computational study

**Fig. 11:**
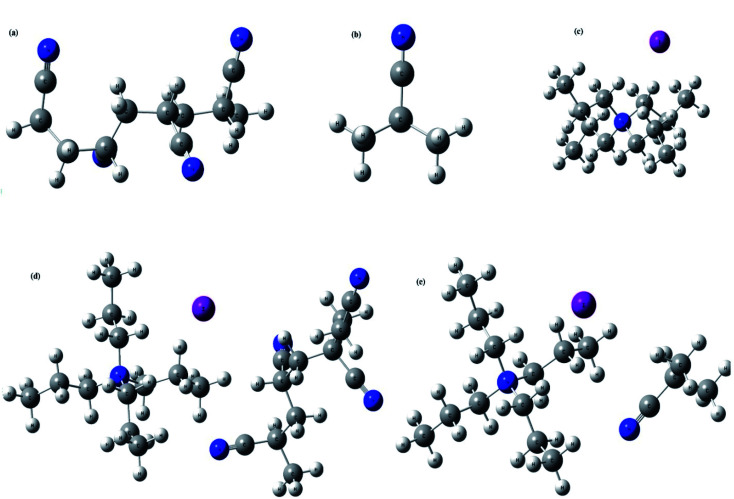
Optimized structures of, (a) PAN, (b) PAN-one, (c) TPAI only, (d) TPAI-PAN, and (e) TPAI-PAN-one.

**Fig. 12:**
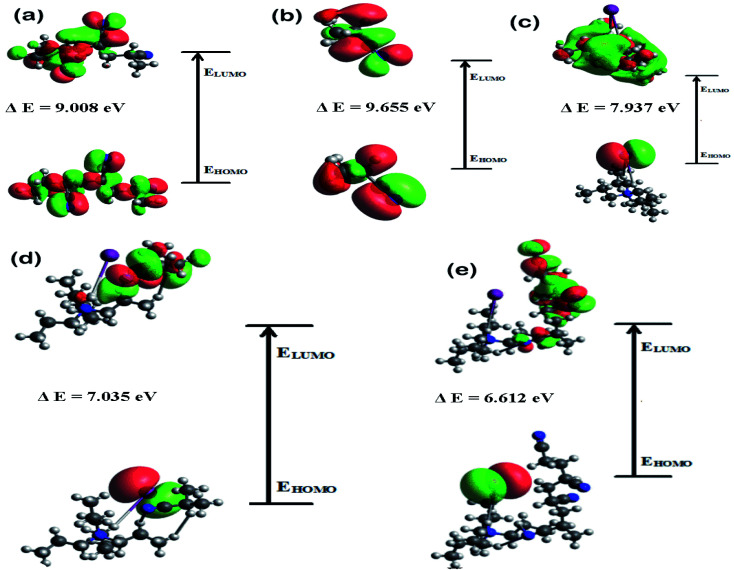
HOMO–LUMO band gap energies of, (a) PAN, (b) PAN-one, (c) TPAI-only, (d) TPAI-PAN-one only, and (e) TPAI-PAN.

A good understanding on the optimized structure with band gap of HOMO and LUMO energy levels is pivotal for the successful application of an electrolyte in a solar cell. Hence, structural optimization and band gap energies of PAN, PAN (one), TPAI-only, TPAI-PAN, and TPAI-PAN-one-only compounds are carried out by using B3LYP/6-31G (d,p) parameters. The optimized structures of these compounds are shown in Fig. 11, and from Fig. 12 the band gap energies of the frontier orbitals are 9.655, 9.008, 7.937, 7.035 and 6.612 eV for the compounds PAN, PAN-one, TPAI only, TPAI-PAN-one-only and TPAI-PAN, respectively.

Several parameters associated with the intra-molecular charge carrying ability, especially band gap energy of the frontier orbitals.^136^ Narrowing the band gap stimulates fast charge transfer rate. From Fig. 12, individual components PAN, PAN-one and TPAI-only show broader band gap than the mixers of TPAI-PAN and TPAI-PAN-one only, which involved with the red shifting of absorption spectra. Besides, the combination of TPAI-PAN exhibits a narrower frontier orbitals band gap (*i.e.*, 6.612 eV) than that of the PAN-TPAI-one-only (*i.e.*, 7.035 eV). Thus, TPAI-PAN has a higher intra-molecular charge transfer ability than TPAI-PAN-one-only electrolyte. From the computational study, in GPE the combination of PAN with 30% TPAI will be the promising electrolytic combination.

### 3.7. DSSC efficiency

The DSSCs with the optimized GPEs were fabricated having the cell structure TiO_2_/N_3_ dye/GPE/Pt and tested. Following the similar trend of conductivity *versus* TPAI concentration, *J*_SC_, as well as efficiency (*η*) of DSSC, increases with the addition of TPAI in the GPEs attaining the maximum of *J*_SC_ (19.75 mA cm^−2^) and *η* (4.76%) for the 30 wt% TPAI, respectively and then, decrease with further addition of TPAI. The *V*_OC_ was also highest (553.8 mV) for 30 wt% TPAI containing GPE.

## 4. Conclusion

The EIS, LSV, FTIR and XRD techniques have been utilized for the characterization of the prepared GPEs. EIS studies showed that the GPE containing 30% TPAI had the lowest bulk impedance and highest ionic conductivity (3.62 × 10^−3^ S cm^−1^). Temperature-dependent ionic conductivity study confirmed that all GPEs obeyed the Arrhenius rule. The 30% TPAI containing GPE exhibited the lowest activation energy. 
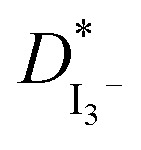
 estimated from the LSV experiments showed that the triiodide diffusion coefficient, 
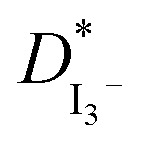
 was maximum with 23.41 × 10^−7^ cm^2^ s^−1^ at 0.051 g I_2_ and 30 wt% TPAI containing electrolyte, which is similar with conductivity results. Exchange current densities (*J*_0_) have been calculated from EIS and LSV measurements, which are reasonably equal to each other. The *J*_0_ is highest for 30% TPAI GPE, which indicated the superiority among the other GPEs. Shifting of FTIR peaks in the GPEs indicates the interaction between PAN and EC/PC. An up-shifting of XRD peak and gradual reduction in intensity followed by diminishing of the peak intensity on continued addition of TPAI in GPEs is evident of the polymer–salt interaction. On the other side, TPAI-PAN based GPE possesses lowest Frontier orbital band gap, indicating the enhanced conductivity leads to maximum efficiency. The DSSC showed the maximum *J*_SC_ (19.75 mA cm^−2^) and *V*_OC_ (553.8 mV) *J*_SC_ and hence highest efficiency *η* (4.76%) for the 30 wt% TPAI containing GPE.

## Conflicts of interest

There are no conflicts to declare.

## Supplementary Material
